# Supplementary material for: Complexome profiling of the Chlamydomonas psb28 mutant reveals TEF5 as an early PSII assembly factor
Source: Plant Cell. 2025 Mar 17;37(6):koaf055. doi: 10.1093/plcell/koaf055 (PMC12164590; doi:10.1093/plcell/koaf055)
Supplement: koaf055_Supplementary_Data [file koaf055_supplementary_data.zip › psb28_interactive.zip!/plots/intensity/qualityPlots/peptides/SIR3_Cre03.g180300.t1.2.html]

### Cre03.g180300.t1.2

Trivial name: SIR3  
SeqCoverage [median]: 4.10  
Mass: 76.61 kDa  
  
 

| Sequence | MissedCleavage | Mass [Da] | wtIntensity | muIntensity | Score |
| --- | --- | --- | --- | --- | --- |
| LYSISSSPLEAPAR | 0 | 1489.78 | 451.95 | 716.30 | 60.43 |
| VQVTVAEVK | 0 | 971.57 | 786.13 | 902.84 | 99.80 |
| VYVQNR | 0 | 777.41 | 255.85 | 0.00 | 86.63 |
|  |  |  | 1493.9 | 1619.1 |  |
